# Supplementary material for: Accuracy of Six Intraocular Lens Power Calculations in Eyes with Axial Lengths Greater than 28.0 mm
Source: J Clin Med. 2022 Oct 8;11(19):5947. doi: 10.3390/jcm11195947 (PMC9572881; doi:10.3390/jcm11195947)
Supplement: Supplementary file 1 [file jcm-11-05947-s001.zip › jcm-1930449-supplementary.pdf]

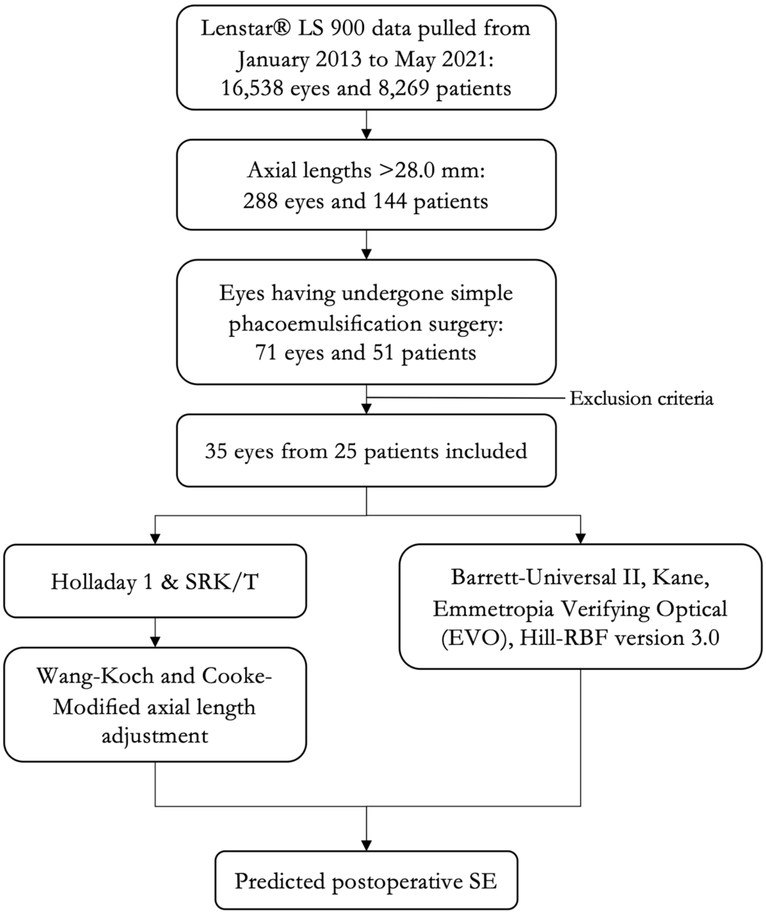

Figure S1: Flow diagram illustrates the process by which patients were selected for the study to obtain the predicted postoperative spherical equivalent (SE) used to analyze the prediction accuracy of the six formulas. The axial lengths were modified with the Wang–Koch and Cooke-modified adjustment prior to their input in the Holladay 1 and SRK/T formulas. The Barrett Universal II, Kane, Emmetropia Verifying Optical (EVO), and Hill-RBF version 3.0 formulas are all unpublished, and the biometric data were input directly into their respective online calculators.
